# Dr. Lallemand's Researches on the Pathological Anatomy of the Brain and Its Coverings

**Published:** 1822-12-01

**Authors:** 


### Medico-Chirurgical Review,

AND

JOURNAL OF MEDICAL SCIENCE.

(Analytical Series.)

"Nec Aranearum textus ideò melior, quia ex se fila fingunt; nec noster vilior, quia ex alienis libamus, ut apes."

Vol. III.] DECEMBER 1, 1822. [No. 11.

### I.

### PATHOLOGY OF THE BRAIN AND ITS APPENDAGES.

*Recherches Anatomico-Pathologiques sur l'Encephale et ses Dependances.* Par F. Lallemand, Professeur de Clinique Chirurgicale a la Faculté de Medecine de Montpellier; Chirurgien en Chef de l'Hôpital Civil et Militaire de la meme Ville, &c. &c. Lettres Premiere, deuxième et Troisieme, 3 vol. 8vo. p. 512. Paris, 1820-21.

"Ars Medica tota in Observationibus."—*Hoffman.*

"Neque enim numerandæ sunt, sed etiam perpendendæ Observationes."—*Morgagni.*

In the 8th Number of this series, we presented the English reader with a comprehensive analysis of Duchatelet's and Martinet's important work on Arachnitis, and we have reason to believe, that the information therein contained, is exciting considerable interest in the profession, and is likely to be productive of much benefit both in diagnosis and therapeutics. The work before us is more comprehensive in its scope, and embraces the pathology of the brain generally. Like that on Arachnitis, it is the patient result of laborious observation—founded partly on materials collected in public hospitals, partly in the private practice of the author and his friends.

M. Lallemand, after some judicious preliminary observations on the difficulty of recognizing, classing, and treating cerebral affections, proceeds to the distribution of his subject, in the following manner.

I. *Affections of the Brain, exempt (as much as possible) from Complication.*

A. Sudden congestion—hæmorrhagic effort short of extravasation, (coup de sang)—with extravasation of blood, (apoplexy.)

B. Inflammation of the brain.

*First stage.* Softening of the brain, with vascular injection, infiltration, or extravasation of blood.

*Second stage.* Softening of the brain, with puriform infiltration, or incipient suppuration.

*Third stage.* Abscess.

C. Chronic affections of the brain; as encysted abscesses—scrofulous tumours—tumours of a fibrous, osseous, scirrhous, or cancerous nature—hydatids—foreign bodies.

II. *Affections of the Tunica Arachnoidea.*

A. Sudden congestion—sanguineous or serous exhalation.

B. Acute inflammation, in different degrees, marked by suppuration, turbid serosity, milky or gelatinous effusion.

C. Chronic inflammation, including thickening of the arachnoid, induration of the same—diminution of its transparency—granulations on its surface.

D. Acute hydrocephalus.

E. Chronic hydrocephalus.

III. *Affections of the Brain and Arachnoid complicated with one another.*

IV. *Diseases of the Spinal Marrow and its Coverings.*

Our author properly observes, that we must not neglect to study the influence of cerebral affections on the symptoms and march of thoracic and abdominal diseases, and vice versa. We should bear in mind, for example, the influence of enlargements of the heart in the production of apoplexy—and the still more remarkable influence of gastric, hepatic, and intestinal affections on the brain; and, of affections of the brain on the functions of the digestive apparatus. It is rarely indeed, that a patient dies of a simple and single affection. Almost always there are several organs simultaneously or successively invaded. Then, as one or other disorder predominates, the others become proportionally obscured. Formerly, (and even still,) the practice too much obtained of examining, after death, only the organ or quarter where the disease seemed to hold its principal seat; and when the *post mortem* appearances did not correspond with the symptoms, the practitioner contented himself with ranging the disorder with the anomalous and inexplicable. The minute attention with which pathological investigations are now carried on, enables us very generally to detect the seat of disease, and appreciate the influence of local affections on the various structures and functions of organs at a great distance from the original focus of these affections. We shall now proceed to follow our author through the divisions and subdivisions traced out in the arrangement above described.

I. *Softening of the Brain, with Vascular Injection, &c.* Of those who have more recently noticed this pathological state of the brain, Recamier, Bayle, Cayol, and Bricheteau, are most conspicuous. The term "softening," (ramollissement) has the advantage of conveying an exact idea of the state of the parts affected, without prejudging the cause of the phenomenon. By "*softening*" of the brain, our author means a kind of liquefaction of *a portion* of its substance, the rest preserving nearly its usual consistence. He lays emphasis on "*a portion*" of the brain; for when we find the *whole* of this organ in a softened state, doubts may be entertained whether it results from disease, or from *post mortem* changes. It is well known, that the brain and spinal marrow very quickly lose their consistence after death, especially in a hot and damp atmosphere, and where the patient has died of dropsy, phthisis, or other disease, that slowly, but greatly deteriorates the powers of life. To form an estimate, without prejudice, of the pathological softening of the brain under consideration, we must have the means of comparing it with other portions of the same brain, where no such change had taken place. M. Lallemand does, indeed, not deny that, the whole of the brain may undergo this change during life; but he rejects these instances from his account, for the sake of obviating objections that might be made against the principles which he endeavours to establish.

The first cases of the disease in question, which have been accurately described, are to be found in that great emporium of facts—the work of Morgagni. The history of Jacoba Zanardi, among others, is very remarkably in point.

*Case* 1. Jacoba Zanardi, of Padua, aged 59 years, was seized with paralysis and fever, for which she was sent to the hospital, where she only survived a few days. On her entrance, although unable to speak, she seemed to comprehend, for she presented her *left* arm to the physician, that he might feel her pulse—the *right* extremities being insensible and motionless. There issued, says Morgagni, much serosity, when the calvarium was removed—the vessels of the arachnoid were injected—there was serous effusion under this membrane, as well as in the lateral ventricles. When the *left* plexus choroides was raised, it was remarked that the thalamus of that side was not of its natural colour, but of a brownish hue.

On dissecting, with the greatest care, all the other parts of the brain, they were found perfectly healthy, except a portion of the left thalamus which was "*extremely soft, almost fluid, and blended with a sanguineous humour, so that it appeared, as it were, rotten. The extent of this altered part was about the size of a large nut.*" All the other parts of the brain were very firm and natural. *Epist.* 5, *No.* 6.

The above case of Morgagni's is certainly very striking. It is evident that he considered the morbid alteration described as a product of inflammatory action, since, he remarks, there was only wanting the fetor, to make the resemblance perfect between it and gangrene. "Nihil nisi gravis odor deesset ut plane *fracidam* pronunciares." The title of this Epistle, it may be observed, is—"Apoplexy which is neither sanguineous nor serous"—and Morgagni's ideas are still more unequivocally expressed in the following sentence:—"Apostema sui generis fuisse hoc credo, agnoscente etiam Avicenna apoplexiam a *repletione apostemante*."*

M. Dan published an inaugural dissertation on apoplexy "considered especially as the effect of inflammation of the substance of the brain," in the year 1807, in which there are several cases related, analogous to the one above quoted from Morgagni. The following short sketches will serve as illustrations of the subject on which we are engaged.

*Case* 2. A mendicant, 78 years of age, entered the HOTEL DIEU in a state of slight apoplexy, after having suffered for some time, an incomplete paralysis of the right arm. The sense of this patient was obtuse, the motions feeble, sluggish, and difficult—especially of the right side of the body; at the same time there was a degree of rigidity in the flexor muscles. He was sometimes somnolent, sometimes in a state of agitation. These symptoms continued the same for some days, at the expiration of which, he lost speech, motion, and sensibility, when death closed the scene.

*Dissection.*—The substance of the brain, for a space of about two inches square, in the posterior inferior pact of the *left* hemisphere, was in a state of putridity (putrilage); the circumference of the disorganized portion being reddened to the depth of two lines. P. 9.

*Case* 3.—Here our author relates a somewhat analogous case that occurred at the Hotel Dieu, under the eye of himself and his friend Dr. Patissier. The subject was a woman

* We wonder that the following remarkable passage, quoted by Bonetus from Willis, (Cerebri Anatom. c. 13,) should have escaped our author, since it is exactly in point. "Cum enim aliquoties cadavera quorundam à *longa paralysi* et gravissima nervorum resolutione defunctorum aperuerim, deprehendi semper *corpora striata præ aliis in cerebro minus firma,* instar amurcæ discolorata, et striis multum obliteratis."—*Bonet. Lib.* I. *Sect.* XV.

80 years of age, who, for a long time, had been troubled in her intellects, and was become morose and irascible. She entered the hospital for a stomach complaint, and was almost always dozing. An emetic was prescribed, which dissipated these symptoms; but they soon returned, and in greater force. Having passed several weeks in a stationary state, she, all at once, lost the senses of hearing and sight, experiencing, at the same time, convulsive twitchings of the *right* arm, and soon after falling into a state of profound stupor. Presently the *right* arm became motionless, but not insensible, the pupils being immoveable. Derivatives of the most powerful kind were tried, but in vain. She died in three days.

On dissection, the vessels of the brain were found gorged with blood. In the middle lobe of the *left* hemisphere, there was found a softened portion of brain—"*the corpus striatum was reduced to a bouillie.*" In the middle of this disorganized portion, some blood was found extravasated.

A case is next quoted from Dr. Abercrombie's paper (case 1) in the Ed. Journal, for July 1818, which is sufficiently in point, and to which our readers can easily refer. In the following case, the morbid structure was found in both hemispheres, yet the symptoms were not the same in both sides of the body.

*Case* 4. A very old woman was brought into the Hotel Dieu on the 15th January, who, according to the account of her friends, had been deprived of sense and power of motion, for fifteen days, subsequent to an attack of apoplexy. (Sinapisms to the feet, and infusion of arnica montana.) Next morning she was more carefully examined. There was paralysis of the whole of the *left* side, with great flaccidity of the muscles and mobility of the joints. On the *right* side, the muscles were in a state of rigid contraction. The face was pale and livid—the mouth slightly drawn towards the left side—the lips, teeth, and tongue fuliginous—pulse hard, small, and frequent—respiration frequent and laborious. She continued three days without any change—on the fourth, subsultus tendinum—and on the fifth, (20th of the disease,) she expired.

*Dissection.* The whole surface of the *right* hemisphere was softened, more deeply in the superior and middle part. This degeneration was about a square inch in extent, and felt quite fluid between the fingers, but without change of colour. About an equal extent of the corpus striatum, of the same side, was reduced into a putrid-looking mass, the colour of lees of wine. This focus was not so exactly limited as in sanguineous apoplexy, but insensibly lost itself in the surrounding parts. Some small portions of the left hemisphere were also softened; but the rest was sound.

This case puzzled our author a good deal at the time of its occurrence; for he had remarked that in all other cases of softening of the brain, there was *rigidity*, or occasional spasmodic twitchings of the paralysed members; whereas, in the present case, there was remarkable *flaccidity* of the paralytic side. Our author's attempt to account for this anomaly may be ingenious, but is not quite satisfactory to our comprehension, and therefore we shall not stop to introduce it here. Unaccountable anomalies will occur in all diseases—and in none more frequently than in affections of the brain.

*Case 5.* The next case recorded by our author was that of a woman, 54 years of age, who had enjoyed good health, but had, for some time, so far lost her sight, that she could not distinguish objects at even a very short distance. Her eyes appeared perfectly sound. On the 1st November, 1817, she lost, all at once, the power of speech; and on the 5th, was carried to the Hotel Dieu. No details of what had passed in the interval could be obtained. The patient understood well what was said to her; but when she wished to return answers, she could only utter inarticulate sounds—she otherwise appeared to be in good spirits. The motion of the tongue seemed free, but the organ inclined a little to the right, when the point was beyond the lips. There was no paralysis of either side—the surface was sensible—the pulse was small, feeble, frequent, and irregular. *Four leeches behind each ear*—in the evening a blister to the nape of the neck. 6th & 7th. No alteration. On the 8th, a laxative glyster was administered; and that day she could pronounce some monosyllables, as *oui* and *non*. On the 9th, complete paralysis of the *right* side, without rigidity. The mouth was drawn to the opposite side. *Six ounces of blood abstracted.* 10th. Total insensibility of the surface of the paralysed side—strabismus. 11th. Same state. 12th. The patient died.

*Examination.* The vessels of the dura mater and arachnoid very much injected. The latter membrane was raised from the pia mater by a serous infiltration between the two. On the anterior lobe of the *left* hemisphere the pia mater was found so adherent (for a space of three inches) to the cortical substance of the brain, that when stripped off, a portion of brain came with it. Opposite to this adhesion the brain was soft as bouillie, in the middle of which softened portion, two small extravasations of blood were found, each the size of a pea. The cerebral substance surrounding these was softened to the middle of the centrum ovale, where it appeared yellow. The lateral ventricles did not contain much serum, but the arachnoid lining them, was covered with very fine granulations. Nothing wrong in any other part of the brain. P. 21, 22.

The gradual development of the paralysis in this patient is very remarkable. First, the sight became affected—then the speech—thirdly, the tongue pointed to one side; and, fourthly, hemiplegia became complete. On dissection, two clots of blood, though very small, were seen; and thus we are led to the first grade, as it were, of apoplexy. The firm adherence of the arachnoid and pia mater to the subjacent cerebral substance, argues, in our author's mind, and fairly we think, that *inflammatory* action had been going on in this diseased portion. It is almost unnecessary to remark that the treatment employed in this case, as in most others in French practice, was any thing but correspondent with the conclusions drawn from the post mortem researches! We are not, however, seeking therapeutics but pathology in the present instance. Our readers well know how to turn to good account, on this side of the channel, the facts which we are now drawing from a distant quarter.

*Case* 6. A widow woman, 54 years of age, had for ten years, experienced great domestic affliction and mental anxiety, at the end of which time, the intellectual functions became disturbed, with debility, general prostration, and almost constant head-aches. On her entrance into the Hotel Dieu, in 1816, her features exhibited the air of great melancholy; and she kept in a sitting posture, as the headache was much augmented by the horizontal position. The respiration was difficult, the pulse irregular and intermitting, the motions of the tongue embarrassed, the muscular force diminished, the extremities benumbed, and the legs and feet œdematous. The arms were occasionally agitated with weak convulsive movements, and a slight subsultus tendinum was felt at the wrists. Gradually the muscular power, especially of the right side, (the diminished sensibility of which, had given place to a sense of formication,) became weaker, and complete paralysis of the *right* side occurred in the night, a month after her entrance into the hospital. The means used were trifling and unsuccessful; and in ten days after this occurrence, the patient experienced violent pains throughout the whole of the paralyzed side, which caused her to cry out piteously. Next day she expired, with tetanic rigidity of the paralyzed side.

*Dissection.* The vessels of the pia mater and arachnoid much injected, and the membranes themselves separated by a gelatinous effusion, like that sometimes seen under a blister. These membranes were easily raised from the subjacent brain, except on the upper part of the *left* middle lobe, where a portion of cerebral substance came away with the arachnoid adherent to the pia mater, of about two inches in extent. The convolutions of the brain, corresponding to this, were flattened, and quite softened, to an inch in depth, the rest of the brain being the firmest our author ever remembers to have seen. In the centre of this softened portion, which was of a greenish yellow colour, was seen a kind of focus or depôt (foyer) the size of a nut, of a dark brown colour, formed by blood, partly extravasated, partly infiltrated, and mixed with the cerebral substance. 27.

Our author thinks it rational to suppose, that chronic inflammation of the arachnoid, by keeping up an habitual congestion in its vessels, terminated in one of those more sudden processes, and incipient hæmorrhage in a portion of the cerebral substance immediately adjacent to the inflamed membrane—and that the presence of this effused blood excited inflammation, or in other words, *softening* of the parts of brain in its vicinity and contact. We have little doubt, indeed, that this softening process is the product of inflammation or congestion of the vascular system of the part.

*Case* 7. The following case* may prove a caution to our junior brethren, not to neglect contusions about the head, particularly in young people. A young man, 17 years of age, of sanguineous temperament, received a blow, by a stone, on the right temple, towards the latter part of June, 1806, which was accompanied by some loss of blood. About the middle of July, he began to complain of head-aches, weakness and pain in the extremities; while his intellectual faculties became considerably disturbed. On the 17th of August, whilst his mother was assisting to dress him, he suddenly lost the faculty of speech, and the power of motion in the *right* side. The pupils were dilated; the paralysed members preserved their sensibility, and were even painful to the touch; the pulse was frequent; and in the evening, there was a febrile exacerbation. In this state, he was carried to the Hotel Dieu, on the 19th of August. On the following days, the right upper and lower extremities became considerably œdematous. On the 30th, he became suddenly comatose by a new attack of apoplexy, in which he lost the use of the *left* side. He died next morning.

*Dissection.* "Three table spoonfuls of bloody serum in the *left* lateral ventricle—some reddened points on the arachnoid covering the thalamus and corpus striatum. Under the anterior part of the *left* ventricle, the substance of the brain, about an inch and a half in extent, was of a deep red colour, the centre of this portion being softened into a kind of semi-purulent condition. In the *right* ventricle was found a clot of black blood, in quantity about an ounce and a half. Under, and behind *this* ventricle, embedded in cerebral substance, was found a clot of black blood, of considerable size. There was some blood extravasated about the commencement of the medulla spinalis." 30.

The above case may give foundation to several useful reflections. In the first place, we observe that, although the contusion was on the *right* temple, the disorganization was

---

* Reported in Dan's Dissertation, before alluded to.

in the vicinity of the *left* ventricle, illustrating, what has been not inaptly termed "*contre-coup*," the symptoms of which were produced about fifteen days after the infliction of the injury. At the end of six weeks, weakness of the extremities was changed into complete paralysis of the right side—*with augmentation of the sensibility, and even of the pain, on pressure.* We may fairly conclude, that the redness of the ventricular parieties, and their reduction into a semi-purulent, soft mass, was the genuine product of inflammation.

Thirteen days later, and we see the *left* side become paralytic; but in this instance, the *sensibility* is lost, and we know that this paralysis owed its origin to *effusion of blood* on the opposite side of the brain. Here then, in the same individual, we have softening of the brain, and apoplexy—two diseases whose symptoms distinguished them during life, and whose pathology we see was sufficiently distinct after death. The hæmorrhage, which ultimately proved fatal, was doubtless determined by the neighbouring congestion and inflammation.

*Case* 8. Passing over several cases, we come to one (13th) presenting some interesting complications.

Anne Benoit, 54 years of age, was of a strong and plethoric constitution, but not very regular in the menstrual evacuations. At each catamenial period, she experienced vertigo and dimness of sight. In 1814, at the age of 51, these symptoms amounted, according to her own account, to a slight attack of apoplexy, but without leaving any paralysis. After this, the cerebral affections became more grave, so that she dared hardly to stoop, without risk of tumbling down. On the 27th of November, 1817, she experienced a more than usual degree of congestion about the head, with loss of sense for a short time. Leeches were applied to the pudendum, and antispasmodics were exhibited by a physician, but without any advantage. On the 28th she entered the Hotel Dieu, and our author found the patient in a comatose state, the eyes closed; but when excited, she could open the *right*, and not the left eye. She answered questions with difficulty, but made it understood that she felt a numbness of the right side, without any muscular power or sensibility to the touch, in the extremities of that side. The face and neck were very turgid and of a livid hue—the jugular veins, on both sides, were as large as a man's finger, and pulsated regularly with the heart; yet the pulse in the arm was feeble and irregular, the action of the heart being remarkably strong, and exhibiting a striking contrast with the pulse at the wrist. There was great pulsation at the epigastrium, and, indeed, throughout the abdomen. Our author instantly opened the jugular vein, without pressure below the orifice, and the blood sprang to a great distance, coming per saltum from the vessel as from a large artery. As the blood flowed, the face lost its violet, and ultimately assumed a pallid hue—the motions of the heart were still irregular and tumultuous, but became more distinct—the pulse at the wrist continuing the same. In an instant almost, five platters (four ounces each) were filled; and when our author stopped the flow, convulsive movements were taking place in the extremities of the right side, and in the muscles of the face on the *left* side. For a quarter of an hour the convulsions were so strong that M. Lallemand could only arrest the hæmorrhage by keeping his finger on the orifice. Even after the convulsions had ceased, he found it a hard matter to stem the effusion. In a little time after the bleeding, the motions of the heart became more regular, and the respiration easy. 29th. The paralysis was complete of the *right* arm and *left* eye. The patient had several epilepti-form convulsions in the course of this day—the state of the face, pulse, and heart becoming the same as before the bleeding. Eight ounces of blood from the feet—the least pressure on the epigastric region renewed the convulsive movements—skin hot and dry. 30th. This was the fourth day of the disease. The face was livid, the lips black, the breathing stertorous, pulse scarcely perceptible, complete abolition of sense, retention of urine. Died at nine o'clock in the evening.

*Dissection.* The tunica arachnoidea thickened, red, and injected. It could be stripped off every part of the brain without being torn. Being well washed, it was found quite opake. The centrical part of the right thalamus was softened to the extent of about half an inch. On the surface of this thalamus, in the lateral ventricle, was a crust of coagulable lymph, or false membrane, which glued it to the septum lucidum opposite. In the *left* side of the brain, several points of the corpus striatum and of the tuber annulare were softened.

*Chest.* Lungs sound. Left ventricle of the heart very much thickened in its parietes, and lessened in its capacity. No stricture of the aortic orifice—dilatation of the root of the aorta into an aneurismal sac, capable of holding the two fists. The origins of the subclavian arteries were materially ossified, puckered, and contracted. Those of the carotids were natural. 52.

In the above case it is interesting to observe that the want of correspondence between the action of the heart, as felt on the chest, and that of the radial arteries at the wrists, is clearly explained by the state of the subclavians at their origins. In all cases, where we have noticed these non-conformities of action, and where we have had opportunities of *post mortem*

examination, we have found *anatomical* reasons for the phenomena; and, therefore, we do not believe that they ever exist independent of such causes. We are aware that Dr. Parry and others advocate the production of such anomalies in the pulse of arteries, by supposing different ratios of momentum in the circulation, without any mechanical obstruction; but we cannot, in our own minds, account for the phenomena in this way.

The aneurism of the aorta must have obstructed the free passage of blood from the left ventricle; and, consequently, kept up a congestion in the lungs, producing the trouble in the respiratory process, and, in fact, causing that turgescence of the face and neck so remarkably conspicuous when the patient was bled from the external jugular vein. It is by no means improbable that, the determination of blood to the head, so long observable in this case, was partly caused by the obstruction at the roots of the subclavians, which might fairly be supposed to produce a greater current through the carotids, which were free in their calibre. It should also be remembered, that the congested state of the venous system of the superior cava, must have kept up a fulness of blood in the head, and thus contributed to the cerebral affection. The appearances in the brain, on dissection, sufficiently account for the nervous phenomena exhibited during life. Lastly, the crust of coagulable lymph on the lining of the thalamus, may lead us to infer, that the corresponding softening of the substance of the same body, was a product of inflammation. The mal-practice of limiting the blood-letting to 20 ounces, under such a state of arterial and venous plethora as this patient exhibited, must be obvious to the veriest tyro in the profession, on this side of the channel.

We shall here introduce the particulars of a case from Bricheteau, which, at first sight, appears not exactly to correspond with the order of things hitherto observed.

*Case* 9. A woman, 34 years of age, had been valetudinary for some time, and subject to wandering pains of the head, &c. On the 21st of March, 1816, she became, all at once, insensible, and continued in this state till the next day, when she was conveyed to the Hotel Dieu. She was then in a profound coma, the head thrown backwards, the eyes fixed and squinting, the pupils contracted and immoveable, the members paralyzed and lying in any direction in which they were placed, little or no sense of feeling, breathing slow and stertorous, temperature and pulse nearly natural. Sinapisms, emetics, &c. without any avail. Died two days afterwards.

*On dissection,* the brain, excepting the tuber annulare, presented no trace of disease. The tuber was reduced to a sort of bouillie. The cerebellum was sound.

Here then there was paralysis of *both* sides, though the lesion in the head was confined to a single spot; but, let it be remembered, that this part was precisely where the fibres of the cerebrum and cerebellum unite in communication with the spinal marrow. We need not wonder, therefore, that the effects of disorganization, in such a place, should be so very general.

Our author closes the narrative or historical part of this letter, with a statement of some cases from Morgagni, which tend to the same point as those which we have already analyzed. He then proceeds to comments or reflections on the facts which he has presented to his readers.

1. In the first place, he observes, that all the cases of "softening of the brain," were accompanied by well-marked vascular injection, infiltration and even extravasation of blood, or a peculiar discoloration. An attentive examination of the cases disclosed various degrees of sanguineous injection, from the most simple distention of vessels, to complete effusion of blood, constituting apoplexy. The slightest degree of injection appears in the brain, where it is *not* softened, and in the membranes of the brain. We often see this injection, or as we should prefer terming it, *turgescence* of the vessels, without any other appreciable alteration in the brain or its coverings. It is then general, uniform, and may, even in a high degree, be sufficient to produce death.* When, under these circumstances, the brain is sliced with a scalpel, we see trickling from the surfaces, a great number of little drops of blood, which are reproduced if wiped away, and give to the medullary substance of the brain, a rosy tint. In cases, where there is softening of the brain, the vascular injection of the sound parts becomes more and more conspicuous as we approach the softened portion—a strong proof of the affinity between softening of the brain and hæmorrhage. The seat of this softening of the brain was found to be far more frequently in the corpora striata, or thalami optici, than in any *one* other part. In fact, the disorganization in question was situated in the abovementioned parts in one half of the whole number of cases—parts where, it will be remembered, apo-

* What, indeed, as we have before remarked on several occasions, can produce a greater degree of pressure on the whole encephalic mass than a general turgescence of its arteries? If it be said that the brain is incompressible, and that there cannot be more blood within the cranium at one time than at another, we answer that, even upon this condition of things, much pressure may be made upon the brain by the *same quantity* of blood when unequally distributed in respect to the two systems of vessels, arterial and venous.

plectic extravasations or exhalations of blood are most frequently observed to be situated.

Our author appears to offer some plausible reasons, why the vascular derangements under consideration should happen more frequently in the corpora striata and thalami, than elsewhere, in consequence of the greater distribution of blood, apparently, to those, than to most other parts of the brain. We should be disposed to attribute this liability, however, in part, at least, to the structure of the brain in those parts, having probably less power of resistance in proportion to the blood-vessels, than other portions of the same organ. But, whatever be the cause, the fact is certain, and ought to be borne in mind when we are proceeding to examine the brain of an apoplectic or paralytic subject. M. Lallemand concludes this letter with arguments and facts to prove, that this "softening of the brain" is the effect of inflammation arrested, perhaps, in its course, by death, *before* purulent suppuration has had time to take place. The following resumée it may not be improper to insert here.

"Sudden cerebral fluxion, congestion, or distention of vessels, is accompanied by vertigo, numbness, dazzlings of the eyes, tinnitus aurium, optical spectra, &c. &c. If this congestion be in a yet higher degree, and the vessels still resist, we have, what may be termed, a 'coup de sang,' (blood-stroke,) with general paralysis, because the vascular turgescence is general; ending in sudden death, or a rapid dispersion of the symptoms. If, in the intervals of these accessions, the vessels remain more or less gorged, we then have a state of habitual somnolency, stupor, and diminution of the intellectual faculties. If the patient be subject to a periodical sanguineous discharge, and this discharge be suppressed, the cerebral congestion generally occurs at the time the said discharges used to happen. The consequences of these repeated fluxionary movements are, habitual dilatation of the vessels and weakness of their coats. If this congestion be in a still greater degree, and concentrated towards a particular point of the brain, where the vessels can offer less resistance, we have hæmorrhage, extravasation of blood more or less considerable, disorganization and sudden compression of the brain, with instant paralysis or apoplexy. If the congestion be less rapid, we have *infiltration*, a kind of combination of blood with the cerebral substance—extravasation of some drops of the same fluid, chronic congestion, softening and disorganization of the brain—a state intermediate between apoplexy and inflammation, and accompanied by slow paralysis and various nervous affections. If the congestion be still more chronic, we have sanguineous injection, symptoms of irritation, convulsions, pain, spasms, &c. with, subsequently, alteration in the substance of the brain, accompanied by numbness, paralysis, first of the upper, and afterwards of the lower, extremities, terminating ultimately in complete cerebral disorganization, with loss of power and flaccidity of the limbs, as in apoplexy.

" A first and sudden congestion leads generally to a sanguineous *extravasation*, with apoplexy and paralysis. After a longer or shorter interval, there are new congestions—the presence of a foreign body augments the irritation—inflammation of the parietes of the sanguineous depôt—consecutive softening of the brain in the neighbourhood. If the clot of blood be not considerable enough to annihilate the functions of that half of the brain where it is situated, we have partial contractions, pain, and convulsive movements of the paralyzed muscles. If the clot be larger, and consequently the compression on the whole of that hemisphere more complete, we have no motions of the paralyzed muscles—the most common case in apoplexies. From this, it will be easy to conceive how an inflammation of the arachnoid membrane, accompanied necessarily with cerebral congestion, may lead to softening of the brain or apoplexy, and *vice versa*, how these latter may determine arachnitis, acute or chronic:—*ubi stimulus ibi fluxus.*" 100.

The *second* letter or volume of M. Lallemand's work, is on "softening of the brain, with *purulent* infiltration, or incipient suppuration," basing his doctrines, as usual, on authentic facts that occurred in the public hospitals.

*Case* 10. On the 1st of April, 1816, a man, 76 years of age, of full habit, was carried to the Hotel Dieu, having been found the preceding evening, in a state of insensibility. Examined at the evening visit, he was observed lying on his back, the left side of the body devoid of feeling and motion, but stiff, and semi-bent. The man often put his right hand to his nose, as if to take snuff—the mouth was half open—tongue dry and black—eyes closed—respiration nearly natural—pulse rather full, but not quick—the intellectual faculties not entirely abolished. Purgative lavements. *3d April.* Very little change, which was also the case on the 5th. On the 6th, died.

*Dissection.* Strong adhesions of the skull and dura mater —the dura mater on the right hemisphere adherent to the arachnoid, which, on that side, was, also, somewhat thickened. A considerable portion of the middle and posterior lobes of the brain was softened, so as to resemble thick pus in every respect. In the inferior portion of the middle lobe, a small quantity of blood was found infiltrated rather than extravasated, giving the brain, in this place, a brownish appearance; the vessels of this part being much dilated, and gorged with blood. The anterior lobe of this hemisphere, and the rest of the brain generally was in a firm and healthy state.*

* It is worthy of remark, that two days before the patient's death, the cold affusion was used, and had great effect in rousing the cerebral

We shall omit M. Lallemand's reflections on this case, as not being particularly interesting; but proceed with facts.

*Case* 11. "In the month of November, 1813, a man, 36 years of age, was carried to the Hotel Dieu, entirely senseless, and without any history of the case. The upper and lower extremities of both sides were so strongly bent, and violently convulsed, that it was impossible for the bye-standers to stretch them out, without using great force. The mouth was half open, the tongue moist, the lips covered with foam, the eyes open and turned upwards, the pupils insensible to light, pulse hard, and so frequent as to be with difficulty counted —whole surface of the body covered with a viscid sweat. M. Lallemand thought, at first, that it was a paroxysm of epilepsy; but having returned several times to the ward, and finding no change at the end of five or six hours, he came to the opinion that it was a case of pernicious or putrid fever, and that the patient would not outlive the night. Sinapisms were, however, applied to the feet, and some antispasmodic medicine was got down his throat. Next day, same symptoms, except that the pulse was weaker. Purgative lavements —more sinapisms. On the third day the convulsive movements had nearly disappeared, being succeeded by subsultus tendinum—the sweats disappeared also—sensibility less obtuse—patient essays to speak a few words. Nevertheless the pulse is weak and quick; the hands, feet, fore-arms, and legs cold; the tongue dry. 4th day. All the nervous symptoms have disappeared; the face slightly yellow; the mouth drier; the extremities still cold—slight stiffness in the members of the left side. 5th day. The skin of the whole body yellow—urine high-coloured and sedimentous; pulse scarcely perceptible. 6th day. Tongue less dry—speech more free—more voluntary motion—patient thinks himself better. 7th day. Patient is cheerful—jaundice diminishing—but the members of the *left* side are paralytic, and at the same time rigid—head always reclining towards the left shoulder—the face a little drawn towards the right side by the contraction of the muscles of the left side. 8th day. In the same state. 9th day. The patient appears much better; is able to walk. When they were going to dress his blisters, he suddenly dropped down, and expired.

"*Dissection.* Much serous fluid between the arachnoid and pia mater on both sides of the head; but more in the right than in the left side. The whole of the cortical substance of the right middle lobe was *softened.* On slicing this lobe, several depôts containing a white and liquid pus were found. The same was observed in the corpus striatum of that side. The cortical substance of the convolutions, and that of the corpus striatum were as white as the centrum ovale. The arachnoid lining the two lateral ventricles, was thickened and granulated on its surface—the ventricles themselves filled

---

functions, and ameliorating, for a time, the symptoms which at length overpowered the patient.

with a lactescent serum. In the chest, all was sound. In the abdomen, stomach sound—liver gorged with blood—gall-bladder filled with thick bile, resembling meconium." 122.

M. Lallemand justly observes, that nothing could be more similar to epilepsy than this case, when first carried to the hospital—the duration of the paroxysm alone distinguishing the two maladies. This epilepti-form aspect he attributes to the arachnoid affection. In the succeeding days, there was a remarkable oscillation in the tide of the circulation, between the brain and the liver—as the latter organ became gorged, and embarrassed in its function, the functions of the brain revived;—and when the determination to the liver subsided, the brain again became oppressed—*vehementior obscurat alterum.* There can be no doubt, that chronic inflammation had long been going on in the arachnoid, especially where it lines the ventricles—and that the *softening* of the brain was the result of an inflammatory process, there can be as little doubt. The sudden and unlooked-for death, when the patient seemed out of danger, is an occurrence, M. Lallemand observes, which very often takes place in suppuration of the brain.

*Case* 12. Joseph Lefebre, 56 years of age, of middle stature and moderately embonpoint, became suddenly deprived of sense, on the 11th July, 1818, and on coming to himself again, was unable to speak, the *right* side being paralytic. A physician prescribed an emetic, leeches to the anus, and a blister to the neck. These means producing no advantage, the patient was transported to the Hotel Dieu, on the 14th July, the third day of his illness. On being received, this man was perfectly collected in his intellects, and the whole surface of the body sensible to the touch. The *right* side was motionless—the lips drawn to the left—the face tumid—the pulse, full, hard, and frequent—respiration and other functions but little disturbed. 15th. The same state. Bled to *twelve ounces*, which was repeated in the evening. 16th. Slight amelioration—the patient pronounced a few words; but the pulse was very irregular. Ten leeches to the neck—sinapisms to the legs. 17th. Pulse less irregular—other symptoms the same. Eight ounces of blood from the foot. 18th. *Has had no motion in his bowels since he entered the hospital.* Bladder gorged with urine—evacuated by the catheter. Eighteen leeches to the neck—sinapisms to the feet. In the evening, prostration of strength—loss of sensibility and muscular power in the right eye—respiration easy—pulse full, strong, and frequent. Twelve leeches to the neck. Died in the night, between the 19th and 20th July, eight days from the commencement of the attack.

*Dissection.* The meninges and substance of the brain were slightly injected. The two hemispheres were examined with the greatest care, and presented no lesion. The cerebellum externally, appeared sound; but, internally, the medullary matter of the *left* hemisphere or lobe, was *softened* and reduced to a bouillie. No lesion in the chest or abdomen." 135.

It is worthy of remark, in the above case, that the intellect was unimpaired till the last moment—a rare circumstance where the inflammation or other lesion is situated in the *cerebrum*. On the other hand, the functions of the heart were greatly disturbed in this cerebellic affection, and the paralysis, as is usual in *cerebral* cases, was on the opposite side of the body. The urinary bladder, however, was paralyzed from both sides we may suppose.

*Case* 13. Mary Lucas, 40 years of age, of sanguineo-nervous temperament, and remarkably corpulent, had a fall in 1814, by which her head was much injured from all accounts. After the wound had healed, this woman became subject to epileptic fits, whenever she was in the least ruffled in her temper. Towards the end of January, 1815, it was observed, that her intellect was impaired, and this affection increased rapidly. On the 1st February, she was conveyed to the Hotel Dieu. Corporeally she appeared in perfect health; but, on examination, in a state of stupor and insensible. The face, which was a little flushed, was occasionally agitated by convulsive movements, as well as the eyes and the *right arm*. When the epigastrium was pressed, these convulsive movements were augmented. The respiration was laborious. *Sinapisms*, *blisters*, *purgative lavements*. Her jaws were so constricted that an emetic could not be administered. During the second and third days, the same stupor continued, and also the convulsive motions of the *right* arm, which were become more frequent and strong. Derivatives were continued; but the patient died in the night, between the 3d and 4th of February.

*Dissection*. "A cicatrice on the scalp over the left frontal bone. The bone beneath was perfectly sound, externally and internally. Near the centre of the anterior lobe of the *left* hemisphere, the dura mater was thickened, and adhered, together with the arachnoid and pia mater, to the cortical substance, which, in this place, was extremely soft, pulpy, and yellow. This alteration of structure had extended to a considerable portion of the anterior lobe. The rest of this hemisphere and of the whole brain was perfectly sound, and exhibited a striking contrast with the diseased portion. No disease in the chest or abdomen." 146.

There can be little doubt, we imagine, that this softening of the brain was the product of inflammation. The accident, the symptoms, all indicate, that such a state had existed. It is, therefore, incumbent on practitioners, to search for, and note, these softenings of the cerebral mass; for we apprehend that many brains have been opened, and reported to exhibit no trace of inflammation, because those effects of inflammation which we are more usually accustomed to, were not obvious. It is only by accurately ascertaining and detecting morbid appearances, and connecting them with the living phenomena, that we can expect to make advances in diagnosis, and, consequently, in therapeutics. We solicit the patient and zealous attention of our countrymen to these points. They may *seem* remote from the objects of immediate practice—but they are not so in reality. The man who studies living phenomena the most closely, and dissects the dead body with greatest minuteness, will be the best practitioner at the bed-side, whether in physic or surgery.

The following case presents several interesting pathological traits.

*Case* 14. "Mary B. 23 years of age, unmarried, entered the Hotel Dieu on the 2d of June, 1814, for a supposed dropsy, having, according to her own account, been tapped for the same, three months previously. The countenance of this young woman was pallid—the ancles a little œdematous—the abdomen too large, but soft, and rather painful on pressure—the skin of the abdomen wrinkled, and of a yellow colour. These last circumstances excited M. Lallemand's attention, and, after much cross-questioning, the young woman confessed that she had been delivered of a child, instead of being tapped for a dropsy, at the Hospice de Perfectionnement, three months before. The yellow colour of the skin, was owing to the repeated applications of cloths wetted with laudanum, to the abdomen, to mitigate pain, which she had felt there for two months past, to which was added a diarrhœa. During the last seven or eight days, there were evening rigors followed by fever—the pulse was small and frequent—the tongue foul—the nervous system rather irritable. *Fomentations, emollient lavements, diluents.* No impression being made on the complaint by these means, an emetic was prescribed. In an hour after taking this, there were great efforts to vomit, but nothing except mucus thrown up. In another hour, convulsions took place, with some foaming at the mouth. Our author was summoned, and found the patient insensible, lying on her back, and the *right* half of the body completely paralytic, the mouth being drawn to the left side. When M. Lallemand went to feel the pulse, he perceived the muscles of the paralyzed arm to be twitched convulsively, and soon after, convulsive movements extended over the whole body. In a few minutes, however, these gave place to complete paralysis again. *Ten leeches to the neck—sinapisms to the feet.* In the evening a similar convulsive accession returned. The succeeding day the respiration was difficult—the prostration of strength great; but the convulsions returned no more. On the day after, or 54 hours from the commencement of the cerebral symptoms, death closed the scene.

" *Dissection.* The arachnoid covering the surface of the brain was opake and thickened—considerable serous effusion between the arachnoid and pia mater. These membranes adhered to the superior and outer part of the *left* middle lobe of the brain, and on being raised, they drew up a portion of cortical substance the size of a nut, which was of a whitish yellow colour, and of little more consistence than that of thick pus. The parietes of the little cavity formed by the detachment of this softened portion of brain, presented the same appearance; but a little farther on, the cerebral substance was perfectly sound. The ventricles contained four or five spoonfuls of limpid serum. *Chest.*—The pleuræ on both sides were thickened, white, and the cavities containing a considerable quantity of turbid serosity. *Abdomen.*—The peritoneum on the small intestines was covered with fine white tuberculated granulations, and the pelvis was filled with a sero-purulent fluid, intermixed with flocculent matters. The mucous membrane of the stomach exhibited a uniform rosy tint—that of the small intestines presented several salient patches, and, near the ileo-cœcal valve, a number of small ulcerations." 154.

It is evident, that this young woman, during or subsequent to her confinement, had contracted a peritoneal inflammation, which became chronic, producing the granulations and sero-purulent effusions observable after death. The diarrhœa was accounted for by the state of the mucous membrane of the small intestines. But it was not the serous membrane in the abdomen alone, that suffered. The same tissues in the chest and in the head, partook of the inflammation; and nothing is more common, especially after parturition, than for a series of the same structures, in different parts of the body, to take on a similar disease. Although there can be no doubt, that disorder was going on in the head, before the administration of the emetic, yet there can be as little doubt, that this injudicious measure hastened the fatal catastrophe, by accelerating the cerebral symptoms—of which, M. Lallemand appears in some measure aware, though he endeavours to obviate the conclusion which one would naturally draw on this occasion.* The following case bears some analogy to the preceding.

*Case* 15. " Mary D. unmarried, 28 years of age, was confined in October, 1819, and did not menstruate afterwards. (The unmarried women in France, after confinement, almost invariably give out their children to nurse, or send them to asylums.) In the beginning of January, 1820, she became affected with severe headaches; and, one month afterwards, having been exposed to wet and cold, when

---

* " Faut-il attribuer le prompt developpment de cette affection à la seule congestion produite par les efforts de vomissement? Il est difficile de concevoir qu'un embarras momentané de la circulation ait pu produire une inflammation, &c. &c." 157.

much heated, the cephalalgia increased—the patient fell into a state of hebetude, and five days afterwards became insensible. Conducted to the HOTEL DIEU on the 23d of February, fifteen days from the increase of the head-ache, she lay in a state of supination and sopor, her face pale and sunken, scarcely sensible when spoken to—puts out her tongue when ordered—abdomen sensible to pressure—screaming and grinding of the teeth during the night. *Sixteenth day.* Rigidity of the members—pulse small—other symptoms the same. *Twelve leeches to the temples and behind the ears—affusions of cold water, during which some degree of reaction was manifested—sinapisms.* From the 17th till the 25th of February, there was no material change from the state above described. On the latter day the patient died.

"*Dissection.* The arachnoid covering the whole surface of the brain, was found opaque and thickened, especially at the base of the brain about the optic nerves and tuber annulare. Some lactescent serosity effused into the net-work of the pia mater. Ventricles contained two ounces of similar liquid. The arachnoid lining the ventricles was covered with evident villosities—the tuber annulare softened and diffluent equally throughout its extent, and of a yellow colour—no sanguineous extravasation. The pleura costalis and pulmonalis on both sides adherent—the lamina of pericardium reflected over the heart, presented two thickened and opake streaks. *Abdomen.* The peritoneal surface throughout, studded with tuberculous granulations in a state of suppuration." 160.

Here, as in the former case, a chronic inflammation of the serous membranes is lighted up by imprudences after confinement. The inflammation of the arachnoid membrane, however, appears to have masked, as it were, the other phlogoses, from its intensity. To it must be attributed the original and obstinate cephalalgia, the subsequent stupor, loss of sensibility, delirium, grinding of the teeth, &c.—the latter symptoms being, indeed, those also of hydrocephalus which, in fact, was present in this case.*

At page 222, *et seq.* our author makes some interesting remarks on the etiology of the class of diseases under consideration. In a few cases, aneurism of the heart very evidently proved an exciting cause of the cerebral affection. Four of the patients were manifestly of the apoplectic form and constitution—and in all these, the invasion of the disease was sudden and severe as in apoplexy. In a considerable number of instances, the patients had previously experienced a

* In this analysis we confine ourselves to those cases which happened under our author's own inspection, and particularly in the public hospitals, for obvious reasons. M. Lallemand quotes several cases from our able countryman, Dr. Abercrombie, and often makes honorable mention of that distinguished cultivator of pathology.—*Ed.*

suppression of some sanguineous evacuation that had become habitual, as hæmorrhoids, epistaxis, &c. The impression of sombre affections of the mind had great influence in developing the disease. Many of the victims had been greatly addicted to the immoderate use, or rather abuse, of vinous and other fermented liquors. All these causes, predisposing and exciting, we need hardly say, are those which lead to apoplexy and inflammation in general—a strong proof that these "softenings of the brain" are the result of an inflammatory process. Let us glance at the influence which this cerebral affection exercises on the other functions of the body.

The *respiration* is remarkably exempt from the influence of the cerebral disorder. In general, this function was calm and regular till within a day or so of the fatal termination, when it became embarrassed, and at length stertorous. This fact may lead us to consider difficult respiration as one of the most fatal symptoms in diseases of the brain.

Nearly the same observations apply to the circulation as to the respiration. The action of the heart is not much under the *direct* influence of the brain; but it is liable to be *secondarily* affected in a considerable degree. Wherever there was trouble of the circulation, it was a symptom of some complication of other disease with that of the brain, as fever, phlogosis in the chest or abdomen, or in the membranes of the brain.

It was before observed, that diseases of the brain appeared to mask diseases of other parts of the body. M. Lallemand does not think, that these other co-existent diseases are checked in their progress, or lessened in their force, by the cerebral affection; but, that the latter renders the symptoms of those other diseases less perceptible to the patient, and consequently less complained of. It is thus that we so frequently see patients allow great accumulations of urine to take place, without making any efforts to void it, in cerebral affections; and even the mucous membrane of the bladder to be considerably inflamed, without their exhibiting any symptom of such occurrence. It is remarkable, also, that this retention of urine, in cerebral affections, augments greatly the original malady; and, therefore, the region of the bladder should always be carefully examined, in this class of complaints, and no accumulations suffered to take place. It is very unfortunate, as M. Lallemand justly observes, that the precursory symptoms of "softening of the brain," like the prodrome of diseases in general, are too often obscure—and that when the symptoms are unequivocal, the disease is too often fatal. This consideration should rather stimulate than paralyze our researches. We are far from being possessed of all the knowledge that may be acquired in our art; and, till that is accomplished, we have no right to cease from our labours.

In a considerable proportion of cases, there was a remarkable alteration in the intellectual functions preceding the invasion of the disease—some becoming impatient, irascible, morose—others melancholy—some exhibiting too high an exaltation of the animal spirits—some experiencing unfounded apprehensions, or spectral illusions, &c. The greater number of patients were previously affected with pains in the head, sometimes of a wandering kind, sometimes fixed, shooting and accompanied by convulsions, morbid sensibility of the retina, pains in the limbs, vertigo, somnolency, stupor, &c. In these latter instances, there was generally found, chronic arachnitis in combination with the softening of the brain. In short, the *precursory* symptoms of this disease, are those which evince a determination of blood to the vessels of the brain, and a morbid exaltation of the excitability of that organ. These symptoms are those, of course, which indicate a disposition to apoplexy or arachnitis; but no harm can result from confounding these three diseases together, in their approach, as the precautionary treatment will be the same. Of all precursory symptoms, the cephalalgia is the most constant; but it diminishes, and at length entirely disappears, as the brain becomes disorganized and incapable of sensation. It was observed, that the intellectual functions became weakened *pari passu*, and in proportion as paralysis affected the extremities.

"Chez presque tous les malades dont je vous ai rapporté les observations, lorsque les membres étaient complètement paralysés, les fonctions intellectuelles étaient comme engourdies; les reponses lentes, tardives, embarrassées, souvent contradictoires; la memoire était chancelante ou entierement abolie; la figure avait perdu toute expression, et portait l'empreinte de la stupeur." 247.

The symptom most remarkably characteristic of softening of the brain, was a contraction of the flexor muscles of the limbs. Sometimes this amounted only to a simple rigidity of the members; at others, it was carried so far that the patient's fist was kept rigidly applied to the shoulder, and the heel to the buttock. When an attempt was made to stretch these members, there was a resistance which it was found impossible to overcome, without offering a violence inconsistent with prudence. The muscles of the face partake of this spasmodic state, and, contrary to what takes place in apoplexy, the mouth is drawn towards the paralyzed side. But we cannot follow M. Lallemand through all his minute symtomatology. We shall proceed to state the particulars of a few cases, where there were strong marks of the disease in question, but where the patients were happily saved.

*Case* 16. " Megnhyel, of strong constitution, much addicted to drink, had, more than once, shewn symptoms of mental alienation, and occasionally, for some days at a time, he experienced a numbness of the upper and lower extremities of the *right* side. On the 18th October he committed, as usual, a debauch in drink, and during the night felt pains throughout all the body, attended with shiverings. He got up several times in the night to drink cold water. In the morning he was found insensible; and at four in the afternoon presented the following phenomena to M. Lallemand: viz. coma profound—mouth very much drawn towards the *left* side—abolition of intellect—diminution of sensation, particularly in the *right* side—spasmodic contraction of the muscles, especially of the *right* side—trismus—pulse full, hard, and frequent—breathing natural. *Bled from a large orifice to the extent of 20 ounces at least—26 leeches to the left side of the neck—sinapisms. In a few hours afterwards 24 leeches in addition were applied—ice to the head.** Next day little or no change. Twenty-four leeches—continue the ice and sinapisms. The application of the ice seemed to restore sensibility. In the evening the breathing was embarrassed. In addition to ice to the head, blisters are to be applied to the insides of the legs. *Third day*. Return of sensibility, sight, and intellectual functions—rigidity of the members continues—some voluntary motion returns. From this time he rapidly convalesced, and soon recovered."

*Case* 17. " In the beginning of January, 1814, there was carried to the Hotel Dieu, a man about 24 years of age, of strong constitution, who had complained, the evening before, of violent pain in the head, for which hot mulled wine had been given him. He had a very restless night, and next morning was found in a senseless state. He was conveyed to the hospital, and the following symptoms were noted:—All the limbs rigidly flexed, with occasionally convulsive agitations—some signs of sensibility when the *left* side was pinched—none when the *right*—mouth drawn to the *right* side—eye-lids shut—eyes turned upwards and divergent—pupils contracted—intellectual functions apparently abolished—pulse slow and soft—face slightly flushed. The physician in attendance was uncertain whether the symptoms indicated cephalitis or a " pernicious fever," and therefore, by way of steering a middle course, ordered twelve leeches to the neck, and if these produced any good effect, to bleed from the arm in the course of the day—if not, to administer tonics and powerful antispasmodics. The leeching produced no effect, *pro or con;* and M. Lallemand (who appears to have been the physician's assistant) found the patient, at two o'clock, in the state above described. From the dissection of former patients with

* This was bold practice for a French physician; and the safety of the patient was probably owing to this comparatively bold step. *Ed.*

similar symptoms, and there finding cerebral inflammation and even suppuration, our author judiciously took it on his own responsibility to open a vein, notwithstanding the pulse was weak and slow, and no good had resulted from the leeching. He therefore made a large opening in the brachial vein, and rapidly abstracted between twenty and thirty ounces of blood. This prompt and copious evacuation produced a remarkable change. The patient opened his eyes—voluntarily extended his *left* arm, and held forth his hand when ordered by M. Lallemand; but could not speak. The *right* arm was less rigid, but still bent—the pupils less contracted. In the course of an hour M. Lallemand returned, and found the patient had fallen back into his former condition of stupor. He therefore determined on re-opening the vein, and drawing off sixteen ounces of blood. This bleeding produced the same striking effects as the former, but in a greater degree. Nevertheless, about nine o'clock in the evening, the original symptoms had nearly all returned; and the pulse had risen in strength. Twelve leeches were applied to the neck, and cloths wetted with cold water were kept constantly applied to the head. Towards eleven o'clock that night, M. L. found the patient asleep, and talking to himself distinctly. The right arm was still stiff; but, when pinched, the patient awoke, and turned himself about. *Sinapisms to the legs.* Next morning the patient was found sitting up, demanding the reason of his being in the hospital, and soliciting something to eat. In the course of the day he walked about the ward, and from this time rapidly recovered."* 295.

---

* We have seen several cases which induce us to believe, that *venous congestion* of the meninges of the brain has a very striking effect in producing *softening* of that organ, as well as sudden death. We shall only state one instance here, but it is a very remarkable one. On Monday, the 9th of September last, we assisted Messrs. Bagster and Pretty, very intelligent surgeons, of Mabledon Place, in examining a fine child, three years of age, who died the evening before, in a very sudden manner. On Saturday, the day before his death, he appeared in perfect health. On Sunday, he had a smart attack of bowel complaint, and the mother sent for Mr. Pretty about 4 o'clock. The child had then such a peculiar look about the eyes and face, and such an apparent fulness about the head, that he expressed to the mother an apprehension that the child would have a fit. Some leeches were ordered to the head, and Mr. P. intended to return in the evening. A little after seven o'clock, however, the boy was seized with strong convulsions, and died at eight o'clock.

We minutely examined him next day. In the course of our lives, we never saw such a state of *venous congestion*. The whole of the veins of the pia mater appeared as if forcibly injected with a strong solution of indigo, and the sinuses were gorged as if ready to burst. The arterial system was almost undistinguishable—what arteries were visible being quite empty. There was no effusion any where; but the brain was so soft, though the body was only a few hours dead, that it would scarcely bear dissection. The net-work of vessels on the tuber annulare, completely excluded from view, the white substance of that part, till it was with difficulty stripped off. The vessels of the plexus choroides were nearly ready to burst. On slicing the brain, *black*, but few or no red points appeared.—*Rev.*

We shall quote but one more case from this second volume of our author, as we find our limits will not permit us to extend our analysis much farther.

*Case* 18. "M. Remy, 60 years of age, had been long affected with gout in the feet, which were quite deformed by its ravages. In working at some theatrical decorations in the beginning of September, 1818, he fell into the orchestra, which, for an instant deprived him of sense, but he soon became collected, and only complained of a slight pain in his side, which went off in a few days. He took to his work, and the accident was forgotten. In a fortnight afterwards, however, it was remarked that Remy was confused in his ideas—that his memory was affected—that he was drowsy—and that his speech was embarrassed. Five or six leeches were applied to the neck. The patient was obliged to take to his bed—the right arm became paralytic, and, from time to time, agitated by convulsive movements—the left hand was frequently applied to his head, or employed in floccitation. The friends of the patient having omitted to mention the fall to the physician in attendance, the patient was treated as having an idiopathic fever, till the 9th or 10th day, when M. Lallemand happened to see him for the first time, and, on inquiry, learnt the true history of the case. At this time the patient fell into so long and profound a syncope, that he was supposed to be dead by many of the by-standers. On recruiting, the right arm and fingers were bent so rigidly as not to be overcome; yet the member and that side were insensible to the touch—the eye-lids were closed—the eyes reversed, divergent, and insensible to the light—hearing, and the intellectual functions in general, quite suspended—whole body covered with a cold and viscid perspiration—breathing difficult, quick, and stertorous—pulse imperceptible at the wrist, and scarcely to be felt in the carotids. In this desperate state the man appeared to have but a short time to live. The blisters and sinapisms had produced but little effect on the skin, and were unlikely to rekindle the dying spark. M. Lallemand, therefore, proposed to the physicians present to pour boiling water on the calves of the legs and thighs, while ice should be applied to the head. This proposal was adopted, but with great reluctance on the part of the physicians, as it was considered cruel to disturb the last moments of a man now moribund. The instant the boiling water touched the skin the patient made sudden movements throughout the whole body—the *left* arm became agitated—the eye-lids opened—the pulse became perceptible at the wrist. In half an hour afterwards the water was applied to the thighs, and the effect was still more remarkable. The face assumed some colour—the pulse rose in force and frequency. The ice was now applied to the head for two hours. The patient seemed to revive, and raising his hand to his head, seemed to attempt removal of the ice. It was taken away whenever the skin of the forehead became cold, and re-applied as soon as it again became hot. In the evening there was considerable reaction, as evinced by the resistance of the pulse and the flushing of the face. Ten leeches were therefore ordered to the neck, and the ice directed to be continued to the head through the night. Next morning things wore a more favourable aspect; but the evening brought an exacerbation of the symptoms. Six leeches to the neck, and ice to the head through the night. On the third day there was some sensibility in the skin of the right arm. Ice to the head in the evening. M. Lallemand, being now obliged to be absent for eight days, was surprized, on his return, to find the patient sitting up and eating. He recovered. The eschars produced by the boiling water were very deep, suppurated profusely, and continued open for fifty days. To this powerful counter-irritation and drain our author attributes the complete recovery of the patient from a state that, at one time, was almost beyond hope." P. 300.

After the symptoms and dissections which have been detailed, there is every reason to believe that the case now related would, had it not been for the judicious measures pursued, have exhibited, on dissection, similar appearances to those so often pourtrayed in the course of this article. We are justified therefore in concluding, that the disease under consideration, especially in its earlier stages, is not beyond the resources of our art.

We now proceed to the third letter or volume of M. Lallemand's work, in which the subject of unequivocal suppuration, or *encysted abscess* of the brain is taken up, and illustrated by numerous cases. These cases are, many of them at least, very interesting to the practical physician and surgeon, their value and importance being quite independent of any doctrine for the support of which they may be brought forward. On this account we shall abridge as many of them as our limits will allow.

*Case* 19. (From Ducrot's Essay on Cephalitis, 1812.) " M. A. about 60 years of age, had a portion of the *left* frontal bone depressed by a violent blow of a stone, by which blood was lost, but which did not deprive him of the power of returning to his house. On the following day he had pulsative cephalalgia, some affection of the memory, inability to move the tongue freely, feeble pulse, depression of energy. Yet his answers were correct. *An emetic.* Third day, deglutition difficult, thirst, heat of skin, frequency of pulse. Fourth day, drowsiness, but still answers coherently. A blister to the nape of the neck. Fifth day—soporose—loss of speech, but appeared to understand what was said—involuntary discharge of urine. Sixth day, sopor still more profound—other symptoms the same. Seventh day, same state. Eighth day, delirium, insensibility, convulsive movements in his limbs and body, with distortion of the mouth and eyes, renewed every quarter of an hour. In the intervals the breathing was difficult and stertorous, with fixed eyes and gaping mouth. Ninth day, the convulsions ceased at midnight, the sopor diminished, and sensibility returned—but the memory and judgment were affected—incipient paralysis of the *left* arm and leg. Tenth day, complete paralysis of the said members, with slight rigidity and pain when they were moved by the by-standers—aspect idiotic—answers vague—optical illusions—a convulsion during the night. Eleventh day, entire loss of sense, and of voice—general immobility—coma—breathing high and difficult—died in the evening.

" *Dissection.* Two inches in extent of the frontal bone was depressed about two lines below its proper level. The arachnoid covering the whole convexity of the brain was thickened, whitish, and lined, on its inner surface, by a layer of albuminous matter. At the basis of the *right* middle lobe of the brain there was a portion of cerebral substance reddened and evidently inflamed." 327.

Ducrot, who relates this case, very clearly shews the connexion of the symptoms with the *post mortem* appearances, but seems not to entertain the least doubt about the propriety of the practice adopted—namely, the Hippocratic treatment of inflammation of the brain and its coverings by—what? —a careful record of its progress, without insulting the omnipotence of Nature by interfering with her ways and means; a precious practice truly! Here M. Lallemand extracts several cases from Morgagni of abscess in the brain, and softening of its substance; but these we shall pass over, as Morgagni's work will soon be in an English dress, that will render it of easy access to the profession at large. The following case, which occurred at the Hotel Dieu, illustrates the effects of the pathemata on the cerebral functions and structure.

*Case* 20. " Juctant, a man about 55 years of age, of small stature, but plethoric constitution, having lost a sum of money constituting his whole property, fell into a state of melancholy, which was succeeded by severe head-aches, profuse perspirations, and fever, accompanied with evening exacerbations. In about a fortnight after this, it was noticed that he was delirious—that he talked incessantly, and always of his pecuniary losses. In five days more, the *left* arm was observed to be paralytic—and this paralysis had continued three days, when he entered the Hotel Dieu, on the 13th January, 1821. His face was then red; the pulse strong, but not frequent; the *right* arm agitated by convulsive movements—the members of the *left* side devoid of sensation and motion, the forearm being bent on the arm;—constant muttering to himself. *Venesection—lavement—diluents.* Next day same state. Venesection repeated more copiously. Fourth day from entrance into hospital, great depression—mouth drawn to the *right* side—insensible—cannot speak. *Sinapisms, purgative lavements.* Sixth day, pupils dilated—stertorous respiration—died at nine o'clock.

"*Dissection.* Difficulty in separating the cranium from the dura mater, and much blood effused from the torn vessels. Arachnoid and pia mater exceedingly injected. In the middle of the *right* hemisphere a purulent depôt was discovered, containing about two spoonfuls of a yellowish green pus—parietes of the depôt converted into a rotten and soft substance, forming a contrast with the rest of the hemisphere—ventricles contained a small quantity of serosity—no other alteration in the brain or other organ." 356.

We have seen cerebral inflammation follow blows on the head at such long intervals that we think practitioners cannot be too much on their guard in such cases. They should watch patients for some months after accidents of this kind. The following case will serve to illustrate the force of the caution.

*Case* 21. "Riom, 17 years of age, had the *right* parietal bone grazed by a musket shot at the battle of Brienne. He was rendered insensible for a short time, but was dressed in haste and sent on to Paris. He arrived at the Hospital of Invalids on the eighth day of the accident. The wound was nearly cicatrized, but the man was tormented by an acute head-ache, lancinating pains in the cicatrix, and constant drowsiness. The integuments being incised, the trephine was applied, and several fragments of bone and of ball were extracted. He was instantly relieved—the wound soon closed—and in three weeks from the operation the patient was discharged from the hospital. In a fortnight after this the head-ache returned, with rigors and fever. *Pediluvia and low diet.* During the next eight days there was almost constant somnolency, with a sense of formication and numbness of the upper and lower extremities of the *left* side; embarrassment of speech; slight constriction of the jaws. The patient entered the Hotel Dieu on the 5th of July, 1813. He could then scarcely hold any thing in his left hand; and was hardly able to walk in consequence of the weakness of the left leg—pulse slow and full. *Venesection, purgative lavement, mustard pediluvia.* 6th. Complete paralysis of the left side—cicatrix inflamed, tense, and painful, with a pasty feel of the scalp. The integuments were again laid open, the bone exposed, and the trephine again applied. The dura mater was very red, covered with fleshy granulations, and extremely sensible to the slightest touch. It was divided by a crucial incision, and the spinal artery bled profusely. No pus was found under the dura mater. *M. Dupuytren suspecting a more deeply seated abscess, plunged his bistoury about an inch into the substance of the brain, and some pus was observed on the blade of the instrument. The bistoury was then plunged a little farther in, and a table spoonful of greyish and somewhat fœtid pus followed. The patient was instantly deprived of speech, and convulsive motions took place in the non-paralysed side.* The dressings, in a few minutes, were filled with blood. An instrument was introduced to compress the spinal artery. The convulsions were increased, the pupils became dilated, the tunicæ conjunctivæ of the eyes greatly injected, the eye-lids became ecchymosed, the same extending to the temples. In the evening the convulsions became less violent; but the pulse was more irregular and embarrassed. Bled from the feet—purgative lavement. At midnight the convulsions ceased, but the man died at one in the morning.

" *Dissection.* A fissure was discovered in the parietal and temporal bones extending to the base of the skull. The dura mater was very red throughout the whole surface of the *right* hemisphere—the arachnoid was equally red, but to a limited extent around the wound. Both membranes on the *left* side were much less injected than on the *right.* Around the incision made into the brain by the bistoury, the cerebral substance was softened to the consistence of bouillie, apparently rotten, and exhaling a fetid odour. On this side of the brain no distinction could be observed between the cortical and medullary substance. The focus of the abscess was seated close to the right ventricle, and the cerebral substance surrounding it was in the soft and putrid state above described, which gradually lost itself in the surrounding sound parts. Another and smaller abscess was situated below the former, and exhibited the same appearances." 361.

The above case is remarkable, not only for the phenomena, and appearances on dissection, but for the boldness of Dupuytren in plunging his knife an inch into the brain in search of an abscess. It was fortunate for his reputation that an abscess *did* exist, and that he *did* happen to strike it! His example, we apprehend, will not often be followed. Our author has seen this puncture of the brain performed five or six times, under the most favourable circumstances, but in all of them without success. " Il ne faut donc pas se faire illusion sur le succès qu'on serait en droit d'en attendre, d'après l'assurance avec laquelle en parlent les auteurs. Quand on a rencontré l'abcès, on se felicite, on croit le malade sauvé; mais on ne tarde pas a voir les symptoms reprendre une nouvelle intensité, et l'on finit par avoir la douleur de le perdre."

From Ducrot's Essay on Cephalitis we shall abridge another case resulting from external violence.

*Case* 22. " Louis Motel, 48 years of age, hurt his head against the mantlepiece of a chimney, without experiencing any thing at the time except a slight sense of stunning. In fifteen days afterwards he felt a degree of weakness in the *right* arm, which gradually increased. The same took place in the lower extremity of the *same* side. Soon after this the intellectual functions were disturbed—pulse feeble and concentrated. On the eighth day (from the commencement of the symptoms, and three weeks from the accident) there was complete paralysis of the right side, accompanied with rigidity and pain. On the fifteenth day some somnolency, change of features, thirst, heat of skin, frequency of pulse. Eighteenth day, loss of sense, involuntary discharges of urine and fæces. Twentieth day, the patient died.

" *Dissection.* In nearly the centre of the middle lobe of the *left* hemisphere a purulent depôt was found, four lines in diameter; the parietes of which were of a dark red colour to two lines in depth. Nothing wrong in any other part of the brain." 387.

We shall analyse but one more case, and then close this article. It is detailed by M. Vaidy, an observant and accurate pathologist.

*Case* 23. " A soldier, 26 years of age, was seized all at once, and without any assignable cause, with a sense of formication in the fingers of the *left* hand, and, at the expiration of 24 hours, lost completely the power of that member. On the fourth day, he complained of pain in his head, with convulsive twitchings, prickings, and formication throughout the *left* side of the body generally, followed by hemiplegia of that side. On the 13th day, motion was recovered in the left hand: and the day following, in the fore-arm and shoulder, the head-ache diminishing. On the 20th day, the power of that side was nearly restored, and the patient could walk. On the 32d and 33d days he had violent convulsions, with return of the cephalalgia and of the hemiplegia. In the succeeding days there was drowsiness at intervals. On the 50th day the patient died in a state of tranquillity. The intellectual faculties, during the whole of this period, suffered no interruption.

" *Dissection.* On examining the brain, there was found in the middle lobe of the *right* hemisphere a purulent collection of about three ounces of homogeneous pus without smell, and of a yellowish colour. The parietes of this abscess were of a deep yellow tint, and plentifully lined with purplish granulations." 412.

We have now presented our readers with ample specimens of the facts contained in M. Lallemand's three letters; but shall not enter into his diagnostics. It is sufficient for us, and for most of our readers, we imagine, to know *when* we have inflammation or abscess in the brain or its membranes, without attempting any very minute distinction as to the *precise* part inflamed or suppurated. The facts, we repeat it, contained in this work and in that on arachnitis, lately reviewed in this Journal, are very valuable in themselves; and when our junior brethren have doubtful cases of cerebral affections under their care, they will do well to read over the collection of cases which we have here, and in a late number, presented them. We beg M. Lallemand to accept the homage of our profound respect and esteem.

